# TDat: An Efficient Platform for Processing Petabyte-Scale Whole-Brain Volumetric Images

**DOI:** 10.3389/fncir.2017.00051

**Published:** 2017-07-31

**Authors:** Yuxin Li, Hui Gong, Xiaoquan Yang, Jing Yuan, Tao Jiang, Xiangning Li, Qingtao Sun, Dan Zhu, Zhenyu Wang, Qingming Luo, Anan Li

**Affiliations:** ^1^Collaborative Innovation Center for Biomedical Engineering, Wuhan National Laboratory for Optoelectronics, Huazhong University of Science and Technology Wuhan, China; ^2^Britton Chance Center and MOE Key Laboratory for Biomedical Photonics, School of Engineering Sciences, Huazhong University of Science and Technology Wuhan, China

**Keywords:** petabyte, terabyte, massive imageset, neuron reconstruction, software platform

## Abstract

Three-dimensional imaging of whole mammalian brains at single-neuron resolution has generated terabyte (TB)- and even petabyte (PB)-sized datasets. Due to their size, processing these massive image datasets can be hindered by the computer hardware and software typically found in biological laboratories. To fill this gap, we have developed an efficient platform named TDat, which adopts a novel data reformatting strategy by reading cuboid data and employing parallel computing. In data reformatting, TDat is more efficient than any other software. In data accessing, we adopted parallelization to fully explore the capability for data transmission in computers. We applied TDat in large-volume data rigid registration and neuron tracing in whole-brain data with single-neuron resolution, which has never been demonstrated in other studies. We also showed its compatibility with various computing platforms, image processing software and imaging systems.

## Introduction

The functions of mammalian brains are based on the activity patterns of large numbers of neural circuits (DeFelipe, 2010; Lichtman and Denk, 2011; Miyamichi et al., 2011; Markram et al., 2015). To precisely reconstruct each neural circuit in the three-dimensional (3D) brain is a fundamental requirement for neuroscience (Lichtman and Denk, 2011; Gong et al., 2016). Mapping these networks requires high-resolution (single-neuron resolution) and large-volume (brain-wide) imaging techniques (Yuan et al., 2015). Techniques for 3D imaging of whole mammalian brains at single-neuron resolution produce several terabytes (TB) to dozens of TB of image data when imaging a mouse brain (Li et al., 2010; Gong et al., 2013, 2016; Zheng et al., 2013), and more than a petabyte (PB) of image data is produced when a primate brain is imaged. As a result, the generated image datasets are too large to be processed by the typical software and computers in biological laboratories. Processing and analyzing these large images have become a challenge (Helmstaedter and Mitra, 2012).

Multi-resolution techniques and image block processing are common approaches for overcoming the difficulty of processing large-volume data. As in Google Maps, only the image data in the current view is read at a given time, thus requiring the image dataset to be divided into several blocks and stored in a hierarchical structure with multi-resolution levels. Recently, two innovative tools, Vaa3D-Terafly (Bria et al., 2015, 2016) and Fiji-BigDataViewer (Fiji-BDV; Pietzsch et al., 2015), as well as the commercial tool Amira-XLVolume (FEI, Mérignac Cedex, France), have adopted this methodology for biomedical studies. These tools enable standard computers to process large image datasets (up to 10 TB; Pietzsch et al., 2015; Table 1) by reformatting volumetric data into a hierarchical dataset. However, the reformatting steps involved in these tools take more than 1 month to complete or require hundreds of GB of memory when processing 10 TB of data. As a result, in practice, these tools have difficulty handling dozens of TB of image data, and processing hundreds of TB or PB of image data is nearly impossible.

**Table 1:** Software or tools for large image volume.

Name	Software extendibility	Custom dataset format	Declared largest dataset	Supported computing platforms
Amira-XLVolume	no	LDA	N/A	no
CATMAID (Saalfeld et al., 2009)	no	2D tile	10 TB	no
Fiji-BDV	no	BDV-HDF5	10 TB	yes
Imaris-IMS (Bitplane)	no	Imaris-HDF5	N/A	no
KNOSSOS (Helmstaedter et al., 2011)	no	KNOSSOS-format	N/A	no
SSECRETT (Jeong et al., 2010)	no	N/A	N/A	no
Vaa3D-terafly	no	TMITREE	2.5 TB	no
Our proposal (TDat)	yes	TDat	1 PB	yes

To fill this gap, we developed TDat, an efficient platform for processing PB-scale volumetric images. Using a novel data reformatting method, TDat efficiently reduces processing time and computer memory consumption. We tested the performance of TDat in reformatting and accessing data. We showed two important applications of TDat in neuroscience, including large-volume data rigid registration and tracing long-range projection neurons in the whole brain. We also demonstrated its applicability for various computing platforms and its compatibility with general image processing software and various imaging systems. The results indicate that TDat is a powerful platform for processing TB- and even PB-scale image datasets.

## Materials and Methods

### Architecture

**Figure 1:**
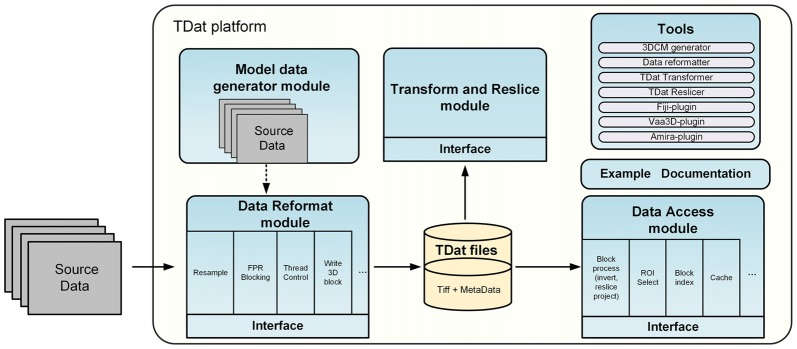
Architecture of the TDat platform. The TDat platform includes four main modules and several tools: the reformatting module, the accessing module, the transform and re-slice module and the model data generation module. These modules provide interfaces for developers. The tools include executable programs and plugins for other bio-imaging software.

The TDat platform includes four modules and several tools. Figure 1 shows the architecture of the TDat platform. The majority of C++ compilers can be used to program with TDat on most operating systems. The TDat platform can be used to develop application programs or plugins for third-party applications. We have also provided executable files and plugins for users who do not have programming skills. For example, the reformatter tool can convert two-dimensional (2D) image sequences into TDat format. The transformer tool can be used to rotate the dataset. The reslicer tool can be used to re-slice the TDat dataset along three orthogonal faces. Users can employ these tools to accomplish specific tasks and can use them as examples to develop other application programs. In addition, we have provided plugins for Vaa3D (version 3.055), Fiji (ImageJ 1.50b, java 1.7.0_09) and Amira (version 6.1.1; FEI, Mérignac Cedex, France). Users can use the TDat datasets in these software programs if they install the plugin.

### FPR Algorithm for Reformatting of the Image Volume

**Figure 2:**
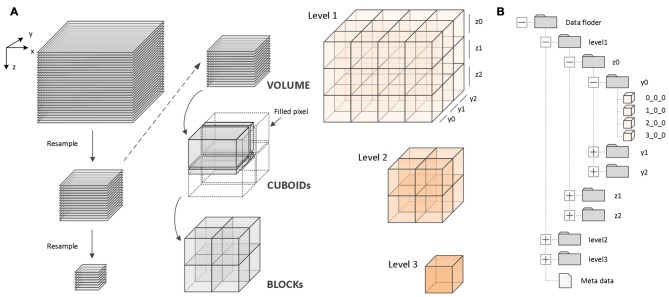
Principle of the fine-grained parallel reformatting (FPR) reformatting method and TDat file organization. **(A)** An example of three-level TDat data to demonstrate the principle of the FPR reformatting method. The image sequence was recursively resampled to create three-level data. For each level of data, the data were split into CUBOIDs and separately read to memory, e.g., level 2 data were split into four CUBOIDs. Each of the CUBOIDs in memory was split into 3D blocks. If the width, depth or length was not divisible by 512, the value was set to 0. X, Y and Z represent width, height and volume depth, respectively. **(B)** An example of three-level TDat file organization. 3D blocks are organized according to a four-level hierarchy of folders by their location in the data space and levels. The 3D block files are named according to x_y_z.

A fine-grained parallel reformatting (FPR) method was used for high-efficiency data reformatting (Figure 2). First, the original image sequences are recursively downsampled with different resolutions. Second, for each image sequence with a different resolution, a region of cuboid data is read into memory (Figure 2A). Lastly, the cuboid data is split into 3D blocks of the same size. The blocks are organized by their location in the data space and levels (Figure 2B). FPR reads the cuboid data (gigabytes (GB) scale) instead of the 2D images (TB scale) for data reformatting. Since the sizes of the cuboid data read into memory are on the order of GB, and the data can be fully split into the 3D blocks, FPR can substantially reduce memory consumption and prevent repeated reading of the same data when data reformatting is performed.

The process includes three main steps. (1) Subsampling: read the original data (level 1) into the computer memory slice-by-slice and then merge two slices and subsample twice to generate a low-resolution image sequence (level 2). Similarly, read level 2 and subsample twice to generate level 3. Recursively generate level 3, level 4 … level n. When the width (X), height (Y) and depth (Z) are less than 512 px, stop subsampling. (2) Cuboid-reading: Read 512 continuous stripes with a total size of WIDTH × 512 px along the Z-axis to generate a CUBOID with a size of WIDTH × 512 × 512 px. If the height of a stripe is less than 512 px, or the slice number (depths) is less than 512 px, assign 0 to the blank pixel. The memory consumption when data reformatting is WIDTH × 512 × 512 × 8 or 16 bit. (3) Block splitting: For each CUBOID block in computer memory, split the CUBOID block into 3D blocks with the size 512 × 512 × 512 px along the X-axis. If the width of a 3D block is less than 512 px, assign 0 to the blank pixel. Write all 3D blocks to the hard disk in TIFF file format, and then free the memory.

The algorithm for data reformatting is as follows:

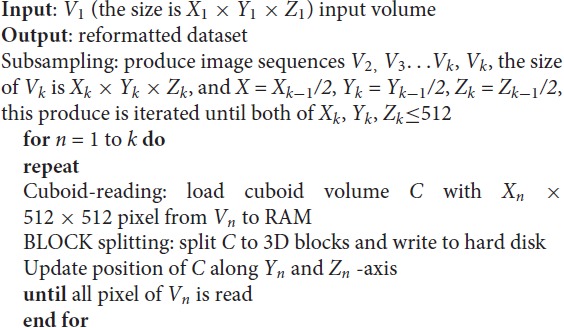


Due to the fine-grained nature of the three steps, memory consumption is low during reformatting. Parallel computing can accelerate these three steps. The speed of the reformatting process can be improved when using an efficient computing platform.

TDat supports 8 bit or 16 bit isotropic or anisotropic image stacks. However, FPR requires TIFF-formatted images because the FPR method requires the image slice to be read in line-scan mode. In this way, TDat can read cuboid data instead of reading whole 2D images during data reformatting. Few image formats (TIFF) support line-scan mode. Therefore, when the input volume is not in TIFF format, TDat will automatically convert them to TIFF format before reformatting, which will increase the total time consumption by 30%.

When data from all levels are reformatted into 3D blocks, these 3D blocks are organized according to a four-level hierarchy of folders based on their location in the data space and levels (Figure 2B). For rapid indexing, the 3D block files are named according to x_y_z rules. For example, for a 3D block that is named 2_3_4, its starting point position in space is (2 × 512 px, 3 × 512 px, 4 × 512 px). Reformatted data also include metadata with “.tdat” as the suffix, which includes a few basic parameters, such as the dataset size, original data resolution, file format, bit depth, level size and file storage location, which accesses the datasets that are required to read and parse the metadata.

### Algorithm for Data Accessing

Each pixel point in the TDat dataset can be accurately mapped to the entire data space. According to the known data space coordinates, TDat can obtain the value of each pixel point from the dataset, which is the foundation of TDat dataset access. We designed two different methods for accessing data.

The first method is sequential reading, which is a simple and efficient means of accessing a TDat dataset in which the 3D blocks are sequentially read and processed, and each 3D block is read separately. This method is suitable for processing the entire dataset.

**Figure 3:**
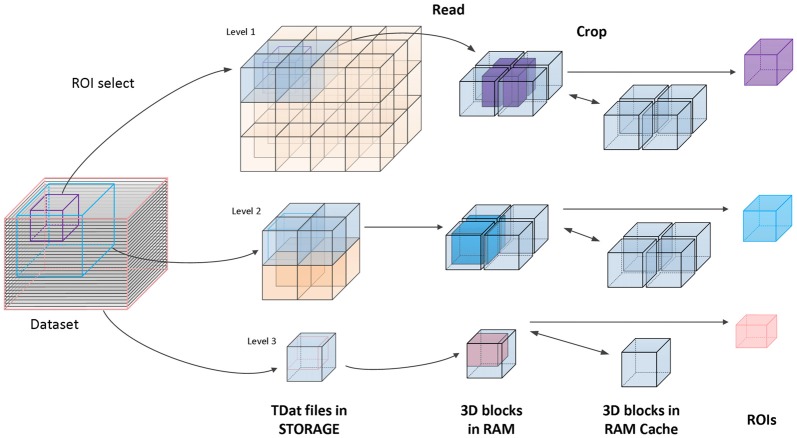
Principle of accessing TDat data. An example of three-level TDat data. When accessing the TDat data, we retrieve different levels of data depending on the size of the region of interest (ROI). The pink ROI is the largest ROI, and we retrieve level 3 data. The blue ROI is medium-sized, and we retrieve level 2 data. The purple ROI is the smallest ROI, and we retrieve level 1 data. The blue transparent 3D blocks need to be read into RAM when calculating the ROI. Subsequently, these blocks are combined according to their positions in the data space, and extra data are removed to generate the ROI data. All of these 3D blocks will cache in RAM. If the 3D blocks that need to be read have been in the RAM cache, they will be read immediately.

The second method is random reading. Random reading reads regions of interest (ROIs) from a TDat dataset. The user defines the position and the size of an ROI; then, TDat provides the corresponding data to the user. The ROIs can be any size at any position in the dataset, which is a common method for accessing a dataset. We use the read-crop strategy (Figure 3) to achieve random reading. The strategy includes the following steps: (1) calculate the 3D blocks that are in the ROI. The level of 3D blocks that needs to be read can be automatically calculated by TDat or can be defined by users. (2) Read these 3D blocks into memory. (3) Combine these 3D blocks according to their positions in the data space and crop extra data to form the ROI data. In practice, random reading is repeatedly called, and the adjacent two calls often use duplicate data. Therefore, we added a memory caching mechanism to accelerate the speed of random reading. We cache recently used 3D blocks in memory. When reading the 3D blocks in step (2), if the 3D block is in the memory, then it can be directly read from the memory. After each random reading call, the memory cache is refreshed using the least recently used algorithm.

### Parallel Computing in Different Computing Platforms

TDat exploits the potential of different computing platforms by parallel computing. We use fine-grained algorithms to improve the degree of parallelism. During data reformatting, each 2D image slice is assigned to a separate thread in the 2D image subsampling process. The data size is on the order of GBs. In the cuboid-reading process, to avoid reading hundreds of large 2D image slices (100 GB or TB scale), separate threads read stripe images in parallel to form a CUBOID (GB or 10 GB scale). After each CUBOID is split, multiple threads write the 3D blocks into storage in parallel in the block splitting process. This process can be executed in parallel because each CUBOID has no duplicate data. In the accessing process, every 3D block file is independent, and the scale is only 100 MB. This process is suitable for parallel reading and calculating. TDat uses two open-source parallel libraries: OpenMP and MPI. OpenMP is used to perform the thread-level parallelism in a single compute node, and MPI is used to achieve parallel task scheduling in multiple compute nodes. Via parallel optimization, the CPU utilization always attains a high level with TDat, and the IO performance tends to become the bottleneck that influences TDat efficiency.

Due to parallel granularity optimization, TDat can be used on almost every computing platform. On a graphic workstation, due to the high performance of the CPU and IO bandwidth (Disk array), TDat uses OpenMP to perform 8–16 thread-level parallel tasks. This application platform of TDat is typical and can handle tens of TB of data. On a high-performance computer (HPC), both OpenMP and MPI are used to implement parallel computing. The performance of TDat can be improved by increasing the number of nodes, and accessing efficiency can be increased using distributed storage. This platform is suitable for hundreds of TB or PB of data.

### Large-Volume Rigid Registration and Re-Slicing Based on TDat

Registration and re-slicing is one of the most important operations in biomedical image processing and visualization (Hill et al., 2001). The imaging dataset is acquired as slices along one of the anatomic planes. When we want to register the dataset to a correct position or identify arbitrary anatomic planes for processing and visualization, traditional software or methods must read the entire dataset into memory to rotate and re-slice the volume, which requires huge memory and time consumption when the dataset is large.

Rigid registration assumes images to be rigid objects that only rotate or translate (six degrees: three rotations and three translations) with respect to one another to achieve correspondence (Hill et al., 2001; Crum et al., 2004). This transformation can be described by a transform matrix. The matrix can be acquired by existing methods, e.g., Amira, ANTS (Avants et al., 2011), etc., using a small-scale (low-resolution) image dataset. Rigid registration based on the TDat method (RRBT) can apply this matrix on a large-scale image dataset.

**Figure 4:**
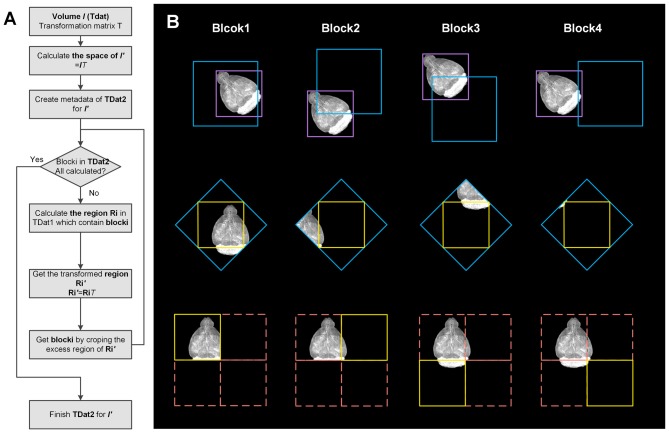
Workflow and illustration of 3D image transformation using TDat. **(A)** Workflow of 3D image transformation using TDat. **(B)** An example of data with a size of 512 × 512 × 512 px was rotated 45° around the *z*-axis. This volume contained only level 1 data with one 3D block after conversion to TDat format. The purple box represents the volume data. After rotation, the size of the new volume was 724 × 724 × 512 px. The new volume required four 3D blocks to store the level 1 data. The pink box corresponds to four 3D blocks. The yellow box represents the 3D block that needs to be calculated every time. The blue box is calculated according to the yellow box’s position and the inverse matrix of rotation. The blue box is read to memory and transformed. The yellow box is created according to the coordinates. The purple 3D block is read four times to finish the transformation; however, the 3D block was read from hard drives the first time and then cached in the memory. The next three times, it was directly read from the memory.

RRBT uses the TDat format to store datasets before and after rotation. The 3D blocks in the after-rotation dataset are separately calculated (Figure 4). Only a small region of the data in the original dataset is read and transformed, which corresponds to specific 3D blocks each time, such as reading a ROI. This process can avoid reading the entire dataset into memory. In this method, however, a small piece of data in the original dataset will be repeatedly read when calculating the two adjacent 3D blocks in the after-rotation dataset, thus wasting a considerable amount of time. The memory caching mechanism of TDat solves this problem.

The rotation process is calculated as follows: (1) according to the metadata of TDat in the original dataset and the rotation matrix, we can calculate the size of the after-rotation dataset. We can also calculate the number of 3D blocks when the after-rotation dataset is converted to TDat format, e.g., the volume data with a size of 1000 × 1000 × 1000 px is rotated by 45° along the *z*-axis; after transformation, the new dataset becomes 1414 × 1410 × 1000 px. The original data split into eight 3D blocks (2 × 2 × 2) on level 1, and the new dataset splits into eighteen 3D blocks (3 × 3 × 2) on level 1. (2) For each 3D block in the after-rotation dataset we can calculate the position of the area that contains the 3D block in the original dataset according to its position in the after-rotation dataset and the inverse matrix of rotation. We can read this data area from the original dataset to memory using random read with the TDat memory caching mechanism. (3) We can rotate the data area according to the rotation matrix in memory and obtain a new data area that contains the 3D block. We can remove the 3D block from this new data area according to the coordinates. (4) Repeat steps 2 and 3 to calculate all 3D blocks in the after-rotation dataset. (5) Other low-resolution level data can be calculated by repeating steps 2–4.

We compared RRBT (rotation by transforming each TDat 3D block) with the traditional method (rotation by transforming the entire dataset). The results (Supplementary Figure S1) showed that RRBT does not affect the accuracy of the rotation.

### Animals and Data Acquisition

Four different datasets were employed. Dataset1 is from a Thy1-EGFP F-line transgenic mouse whose whole brain was imaged using a light-sheet microscope (UltraMicroscope II, Lavision Biotec GmbH). Dataset2 is from a Thy1-EGFP M-line transgenic mouse whose whole brain was imaged using a two-photon fluorescence Micro-Optical Sectioning Tomography system (2p-fMOST; Zheng et al., 2013). Dataset3 is from a Thy1-EGFP M-line transgenic mouse whose whole brain was imaged using a brain-wide precision imaging system (BPS; Gong et al., 2016). Dataset4 is from an Sprague-Dawley (SD) rat whose whole brain was imaged using a Micro-Optical Sectioning Tomography system (MOST; Li et al., 2010). The raw data sizes of the four datasets were 862 GB, 1.17 TB, 2.74 TB and 38 TB, respectively. Details of the four datasets are provided in Table 2. Animal care and use was done in accordance with the guidelines of the Administration Committee of Affairs Concerning Experimental Animals in Hubei Province of China. All animal experiments followed the procedures approved by the Institutional Animal Ethics Committee of Huazhong University of Science and Technology.

**Table 2:** Detail information of whole brain imaged datasets in this study.

Animal		Data acquisition	Raw data size	Pixels	Resolution (μm^3^)	Bit depth	TDat size	Reformatting time^(a)^
Dataset1	Thy1-EGFP mouse	LSM^(b)^	862 GB	14,080 × 19,656 × 1673	0.5 × 0.5 × 2.5	16 bits	648 GB	3.3 h
Dataset2	Thy1-EGFP mouse	2p-fMOST^(c)^	1.17 TB	13,913 × 18,000 × 5115	0.5 × 0.5 × 2	8 bits	561 GB	4.7 h
Dataset3	Thy1-EGFP mouse	BPS^(d)^	2.74 TB	28,452 × 21,866 × 4834	0.32 × 0.32 × 2	8 bits	841 GB	10.3 h
Dataset4	Golgi-stained rat	MOST^(e)^	38 TB	31,724 × 81,200 × 16,203	0.3 × 0.3 × 1	8 bits	15 TB	124 h

*^(a)^The reformatting time for a graphic workstation with 3.1 GHz Intel E5-2687w×2, 192 GB of RAM, a disk array. ^(b)^Light-sheet microscope. The whole brain was cleared by the 3DISCO (Ertürk et al., 2012) method. ^(c)^Two-photon fluorescence micro-optical sectioning tomography system. ^(d)^Brain-wide precision imaging system. ^(e)^Micro-optical sectioning tomography system*.

### Model Dataset

To test our methods, we designed a physical model dataset, which is referred to as the 3D chessboard model with Gaussian noise (3DCM) for benchmark datasets (Supplementary Figure S2). The 3DCM used an N slice TIFF sequence in which the size of each slice size was N × N px, each TIFF formed a 256 × 256 px black and white grid, and the black and white colors changed every 256 slices. To simulate the compression ratio of the image files, we added 5% Gaussian noise to the images. We provided a program for generating 3DCM model data that could automatically generate a continuous image sequence. We generated several 3DCM model datasets with different sizes for benchmarking (100 GB to 1 PB). Details of the model datasets are provided in Supplementary Table S1.

### Environments for Benchmarking

Two computing devices were used: a graphical workstation and a computer cluster. The workstation was equipped with 16 cores, 192 GB of RAM, and a disk array. The computer cluster contained 20 nodes. The nodes were equipped with 20 cores, 128 GB of RAM, and 10 GB Ethernet. All nodes were connected with a Lustre file system. Details of the computer configuration are provided in Table 3.

**Table 3:** Detail information of the computers configuration in this study.

	CPU	RAM	Storage	OS
Workstation	Intel E5-2687w×2 3.1 GHz	192 GB	DELL MD1200 48 TB disk array RAID0	Windows 7 Professional
HPC Cluster (20 nodes)	Intel E5-2660 V3×2 2.6 GHz	128 GB	Inspur Lustre file system 3.08 PB^(a)^	Linux Red Hat 6.3

*^(a)^Each node was connected with the Lustre file system via a 10 GB Ethernet*.

## Results

### The Performance of Data Reformatting

**Figure 5:**
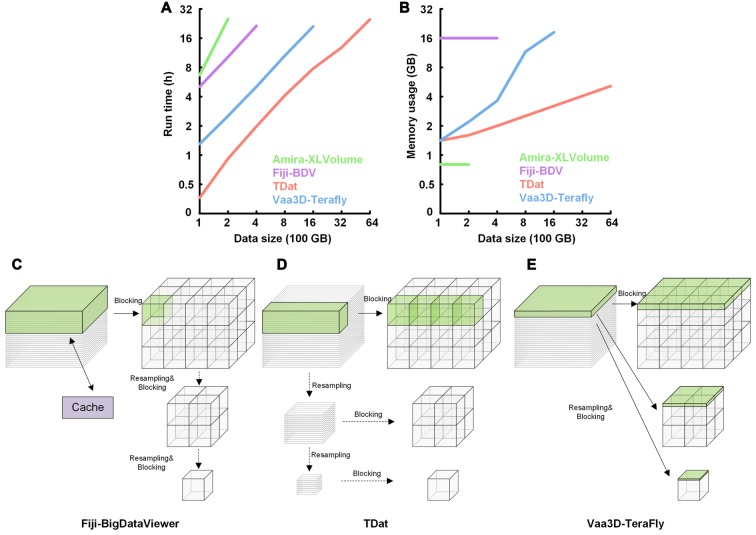
Performance of TDat for reformatting model data and the comparison of the reformatting principle with different methods. **(A)** Run time for reformatting different sizes of model data. The numerical data can be found in Supplementary Table S2. **(B)** Memory consumption for reformatting different sizes of model data. The size of the data for reformatting exponentially increased from 100 to 6400 gigabytes (GB). The numerical data can be found in Supplementary Table S2. **(C)** Principle of reformatting in Fiji-BigDataViewer (Fiji-BDV). The green region in the original sequences represents the data that need to be read into memory to generate a block. One slice is read into memory once and cached. If the cache is out of space, it is updated and the previously read data are removed. **(D)** Principle of the FPR method in TDat. The original sequences were recursively subsampled; next, a CUBOID was read into memory and split into 3D blocks. **(E)** Principle of reformatting in Vaa3D-TeraFly. The green region in the original sequences represents the data that need to be read into memory to generate multi-resolution tiles that are part of the blocks. The slice number of the green region in the original sequences is 2n-1, where *n* represents the number of the multi-resolution level. These slices were read into memory to ensure that one piece of a tile could be generated in the block with the lowest resolution level.

We used TDat to reformat 100–6400 GB of model data on a graphic workstation (Intel Xeon E5-2687w×2/192 GB). The time and memory consumption were compared to the results from Amira-XLVolume, Fiji-BDV and Vaa3D-Terafly, which were used on the same workstation. The results indicated that TDat was 4–32 times faster than other methods and consumed less memory (several GBs; Figures 5A,B and Supplementary Table S2). For 100 GB of data, the memory consumption of TDat was only 1 GB. When the data size increased to 6400 GB, the memory consumption of TDat was only approximately 5 GB (Figure 5B). Memory consumption during data reformatting is linearly associated with the width of the data volume. For example, the size of 6400 GB of model data is 19,012 × 19,012 × 19,012, and the memory consumption is 4.64 GB (19,012 × 512 × 512 × 8 bit). TDat was able to reformat 6400 GB of data per day, and the relationship between computational time and data size was linear (Figure 5A). The memory consumption of Amira-XLVolume and Fiji-BDV remained unchanged when reformatting data of different sizes (1 GB and 16 GB); however, they were only able to reformat 200 GB and 400 of GB data in 1 day, respectively. Vaa3D-Terafly was relatively fast and was able to reformat 1600 GB of data in 1 day. However, the memory consumption of Vaa3D-Terafly was huge; for 1600 GB and 6400 GB of data, it required 18 GB and 92 GB of memory, respectively. Our results suggest that TDat was much more efficient both in time and memory usage during the reformatting of large-volume data.

TDat improves the efficiency of reformatting by using a new strategy for reformatting. It finds a balance between large memory consumption and frequent data reading during reformatting. TDat reads only cuboid data into memory to control large memory consumption during data reformatting, and It uses parallel computing to accelerate the speed of reading/writing data during data reformatting (Figure 5D). Vaa3D-Terafly only needs to read the datasets once to complete the reformatting, but with data larger than several TB datasets, memory consumption will increase sharply (Figure 5E). Fiji-BDV can reformat any size of data with a fixed memory usage, but the dataset is read repeatedly when the size of dataset is large which will increase the time consumption evidently (Figure 5C).

### The Performance of Data Accessing

**Figure 6:**
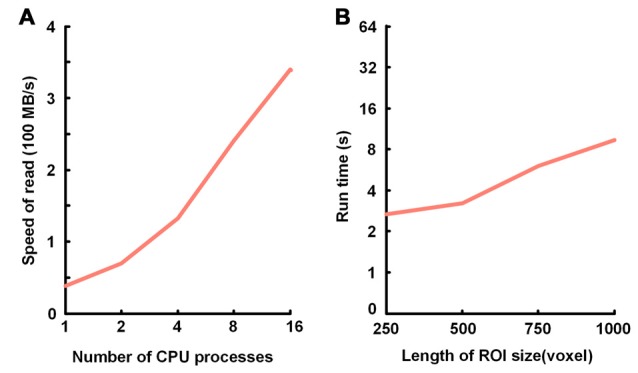
Performance of TDat for accessing model data. **(A)** TDat sequential-read speed (file size/read time) for varying parallelism. The time cost for reading 1728 blocks in 200 GB of model data; the total file size was 118 GB. All benchmarks were repeated three times for each experiment, and the average speed was then calculated. **(B)** The run time for TDat random-read, varying the size of the ROIs. The size of the data for accessing consisted of 6400 GB of model data. The computer platform was a 16-core workstation with 32 GB of RAM and a 44 terabytes (TB) disk array. All benchmarks were repeated 20 times for each ROI size, and then the average run time was calculated.

We verified the TDat performance of data accessing on a graphic workstation (Intel Xeon E5-2687w×2/8 GB). The test included sequential reading and random reading. In sequential reading (Figure 6A), for TDat to adopt parallel reading, each of the 3D blocks is read into memory in parallel so that it can make full use of the bandwidth of the storage to improve the performance of TDat accessing. With a higher parallelism, the speed of TDat accessing was higher. In random reading (Figure 6B), we read four different sizes of cubic data from TDat datasets and measured the time consumption. The sizes of the cubes were 250 px, 500 px, 750 px and 1000 px. The results indicated that the time consumption was only 7 s for reading cube data with a length of 1000 px.

### Whole-Brain Dataset Rigid Registration and Re-Slicing by TDat

We consider Dataset3 (see Dataset3 in Table 2) as an example. This dataset was composed of whole-brain images of a Thy1-EGFP M-line transgenic mouse imaged using BPS (Gong et al., 2016). The original voxel size of 0.32 × 0.32 × 2 μm^3^ was resampled to 2 × 2 × 2 μm^3^ and 0.5 × 0.5 × 0.5 μm^3^; the sizes were 58.9 GB and 3.68 TB, respectively. The smaller-size data were calculated by RRBT and Amira for comparison. The larger-size data were calculated only by RRBT. The transform matrix for rigid registration was obtained by 10 × 10 × 10 μm^3^ resampled data. The transform matrix was

$$\left[\begin{array}{cccc} 0.868588 & -0.391007 & -0.30441 & 0\\ 0.273509 & 0.89055 & -0.363469 & 0\\ 0.413211 & 0.232447 & 0.880467 & 0\\ 0 & 0 & 0 & 1 \end{array}\right]$$

**Figure 7:**
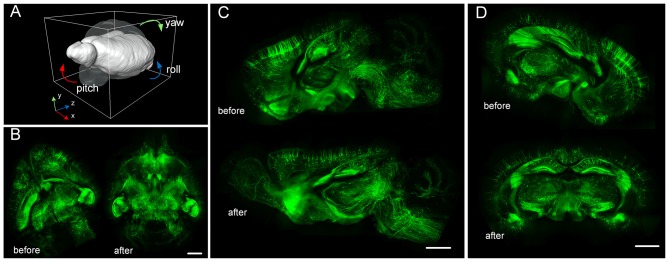
Large-volume re-slicing using TDat. **(A)** The transparent brain indicates the position before rotating; the white brain indicates the position after rotating. **(B)** Horizontal plane maximum intensity projection before and after rotating. **(C)** Sagittal plane maximum intensity projection before and after rotating. **(D)** Coronal plane maximum intensity projection before and after rotating. The dataset is from a whole brain from a Thy1-EGFP M-line transgenic mouse imaged using brain-wide precision imaging system (BPS; see Dataset3 in Table 2). The scale bar is 1 mm; the thickness of the projections is 256 μm.

We used RRBT to transform the dataset to a correct position and to re-slice the dataset to obtain three anatomic planes (Figure 7). RRBT used 2.4 h and only 7 GB memory to finish transforming the dataset with 2 × 2 × 2 μm^3^ resolution on a graphic workstation (Intel Xeon E5-2687w×2/192 GB). However, Amira required 225 GB of memory and could not complete the job. The memory consumption of Amira was far more than what is commonly used. When we changed the dataset to 0.5 × 0.5 × 0.5 μm^3^, Amira could not read such large-volume data into memory to perform transforming. RRBT required only 7 GB of memory to finish transforming and re-slicing three anatomic planes on a graphic workstation.

### Tracing Long-Range Projection Neurons in a Whole-Brain Dataset

The functions of mammalian brains are based on the activity patterns of neural circuits, which consist of local connections and long-distance connections (Miyamichi et al., 2011; Harris and Shepherd, 2015). An increasing number of imaging methods can map long-range connections, which is more difficult than mapping local connections (Ragan et al., 2012; Gong et al., 2013; Zheng et al., 2013). However, long-distance neurons often extend through several nuclei or even span the entire brain (Lichtman and Denk, 2011). The size of the data that contains long-range projection neurons is far greater than usual computer memory size; it is even difficult to read the data into memory before tracing. Therefore, previous methods have difficulty tracing long-range projection neurons.

**Figure 8:**
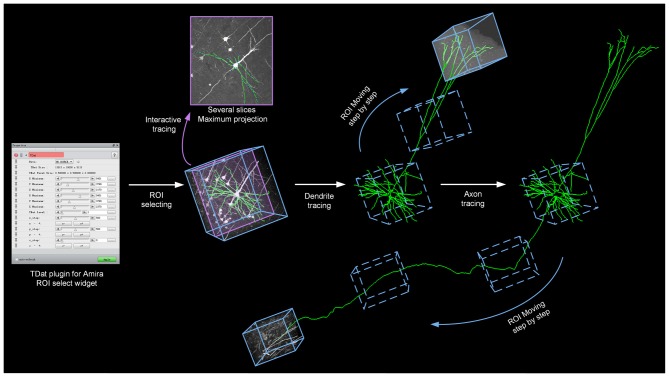
TDat-Amira plugin for interactive tracing of long-projection neuron morphology. The TDat-Amira plugin was used to read the ROI from the TDat dataset into Amira. Filament editor was used to interactively trace the neuron fiber. When this ROI is traced, another ROI is read along the direction of the fiber for continuous tracing. The ROI is a cortex from a Thy1-EGFP M-line transgenic mouse. The dataset was imaged using a two-photon fluorescence Micro-Optical Sectioning Tomography system (2p-fMOST; see Dataset2 in Table 2).

TDat converts large image data into TDat file format and adds a data accessing module in Amira. Users need only 1 day to convert a several-TB dataset on a graphic workstation (Intel Xeon E5-2687w×2/192 GB) and to begin tracing neurons in the whole-brain data. Users can apply the Filament editor module in Amira to interactively trace neurons. We showed how the TDat-Amira plugin can be used to continuously read ROIs from the TDat dataset into Amira and interactively trace long-range projection neurons (Figure 8).

### Application to Whole-Brain Images Acquired by Various Imaging Systems

**Figure 9:**
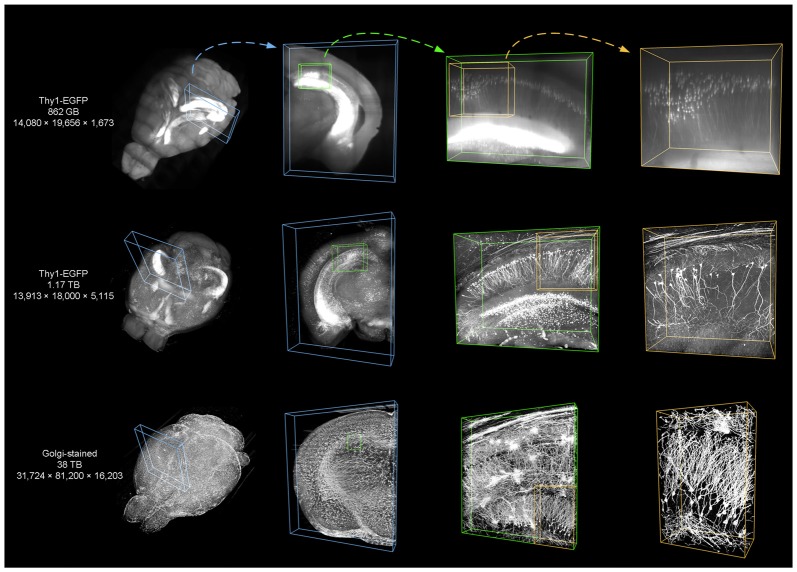
Using TDat to process different sizes of image data that were acquired by different systems. From top to bottom, the three datasets are from the whole brains of a Thy1-EGFP F-line transgenic mouse imaged using a light-sheet microscope, a Thy1-EGFP M-line transgenic mouse imaged using a 2p-fMOST, and a Sprague-Dawley (SD) rat imaged using MOST. The labeling method, raw data size and pixel size are given in the left-most column (see Dataset1, 2 and 4 in Table 2 for details). TDat was used to reformat the data to the TDat file format and access the ROI. From left to right, the size of the ROI continuously decreases, the TDat level decreases, and the resolution increases. The resolution of the right-most ROI is the original resolution of the dataset.

We used TDat on three different datasets (see Dataset1, 2 and 4 in Table 2) to demonstrate the applicability of TDat for various labeling methods and imaging systems and different data sizes (Figure 9). The three datasets included a whole brain from a Thy1-EGFP F-line transgenic mouse imaged using a light-sheet microscope, a whole brain from a Thy1-EGFP M-line transgenic mouse imaged using 2p-fMOST (Zheng et al., 2013), and a whole brain from an SD rat imaged using a MOST (Li et al., 2010). The sizes of these three datasets were 862 GB, 1.17 TB and 38 TB, respectively. We converted the original image data into TDat format and visualized the ROIs. A variety of datasets with different resolutions were read according to the size of the ROIs. When visualizing the whole-brain data, low-resolution data were read. When visualizing single cells or nuclear groups, high-resolution data were read.

### Extending TDat to High-Performance Computers

All studies of big data are inseparable from HPCs. TDat can both process big image data on common desktop computers and be used on HPCs for processing larger data. Due to the fine-grained algorithms of TDat for reformatting and accessing methods, we used MPI to assign computational tasks to different compute nodes. These computational tasks are performed in parallel, which will greatly improve the computing speed.

**Figure 10:**
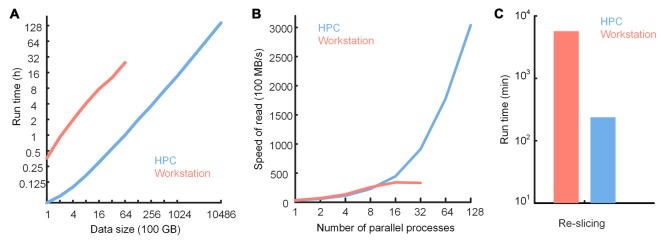
Performance of TDat on a workstation and a high-performance computer (HPC). **(A)** Run time for reformatting different sizes of model data. The data size exponentially increased from 100 GB to 1 petabytes (PB). The numerical data can be found in Supplementary Table S3. **(B)** TDat sequential-read speed (file size/read time) for varying parallelism (for the workstation, parallelism represents the number of CPU cores in parallel; for the HPC, parallelism represents the number of processes in parallel on 20 nodes) on two platforms. The data consist of 6400 GB of model data. When using 16 or more threads to parallel read, the speed of the sequential-read on the workstation was no longer increased because the speed was at the upper limit of the file system. This also illustrates that TDat can make full use of hardware resources. **(C)** Performance of re-slicing on two platforms. The data for re-slicing were the same as the data in Figure 7. The size of the data was 3.68 TB. The details of the computer platform configuration are given in Table 3.

We tested the performance of TDat in reformatting, accessing data and large-volume re-slicing on an HPC and compared performance on a graphics workstation with that on an HPC (Figure 10). The results showed that TDat can be well applied to HPCs and that processing efficiency was improved as expected. When TDat was running on a workstation, the reformatting performance was 6 TB of data per day (Figure 10A and Supplementary Table S3). The efficiency of reformatting with an HPC was an order of magnitude higher than that on a workstation. A 20-node HPC required only 13.6 h and 6 days to reformat 100 TB and 1 PB data, respectively (Figure 10A); memory consumption was only 12 GB and 25 GB for each process, respectively. Processing speed was further improved by adding nodes. In an accessing data test, HPCs could provide larger parallelism and more efficient storage, thus increasing the accessing speed (Figure 10B). The performance of re-slicing on HPC was also an order of magnitude higher than that on a workstation (Figure 10C). TDat provides researchers with more opportunities to select a computing platform according to their practical requirements.

### Extending TDat to Other Image Processing Software

We wrote plugins based on TDat to demonstrate that TDat can enrich existing image processing software to access large-volume data. Here, we show two typical examples: the TDat-Fiji plugin and the TDat-Vaa3D plugin. Supplementary Figure S3 illustrates how the TDat-Fiji plugin can be used to read the TDat dataset into Fiji (Schindelin et al., 2012) and how the TDat-Fiji plugin can be applied to perform image processing and cell counting. Supplementary Figure S4 demonstrates the application of the TDat-Vaa3D plugin to read the TDat dataset into Vaa3D (Peng et al., 2010, 2014) and the application of the Vaa3D to visualize the ROI and the segmentation of neurons. Due to the compatibility of TDat with common image processing software, researchers can use their preferred image processing software to process large-volume image data.

## Discussion

In this study, we invented TDat, a platform for processing TB and even PB-sized volumetric images. TDat uses a novel data reformatting method by reading cuboid data and by parallel computing during the reformatting process, thus reducing the consumption of memory and time. TDat uses a parallel reading and a memory caching mechanism to achieve rapid access to reformatted datasets. It can be used on various computing platforms and is compatible with general image processing software. TDat enables neuroscience researchers to process large whole-brain datasets using existing hardware.

TDat improves the efficiency of processing big data using a new strategy for reformatting. It solves the problem of large memory consumption and frequent data reading during reformatting (Figure 5). A currently available software program, Vaa3D-Terafly, also focuses on reducing frequent data reading while reformatting (one at a time; Figure 5E). The methodology of Vaa3D is particularly well suited to address small datasets, but memory consumption increases sharply with datasets larger than several TB. Another existing software program, Fiji-BDV, strictly controls memory usage (Figure 5C). In theory, Fiji-BDV can reformat any size of data with a fixed memory usage. However, the same dataset may be read repeatedly, which becomes a serious issue when the size of the dataset is large. The computational time of Fiji-BDV increases sharply when the size of the dataset becomes larger.

TDat reads only cuboid data into memory during data reformatting. Its memory usage is linearly associated with the width of the data volume (Figure 5D). When reformatting 100 TB of data, only 12 GB (each process) of memory was required for TDat. To reformat 1 PB of model data, only 25 GB (each process) of memory was required. By comparison, the memory usage of Vaa3D-Terafly was exponentially associated with the number of the multi-resolution levels of data (Figure 5E). In theory, the reformatting methods of Vaa3D-Terafly need several hundreds of GB or TB of memory to reformat 100 TB of data. Although TDat needs to read the original data twice, this process improves the efficiency via parallel computing. Compared with the existing software, TDat is the fastest method for data reformatting, taking full advantage of computer hardware.

In the study of connectomics, other tools are available for handling large-scale images, such as Ssecrett (Jeong et al., 2010), CATMAID (Saalfeld et al., 2009), Knossos (Helmstaedter et al., 2011), DVID (Katz, 2012), and The Open Connectome Project (Burns et al., 2013). These tools are mainly used for processing 3D electron microscopy image data. Most of these tools adopt a client-server architecture that stores the image data on the server side and lets the client request data from the server by APIs (e.g., HTTP request handling). These data, pushed to the client side, are 2D slices or arbitrary cross-sections. However, these methods are not intuitive when users need volume rendering to visualize data for processing instead of 2D slices or arbitrary cross-sections because volume data are usually too large to be transferred between the server and the client. With the progress of imaging technology, acquiring a PB-sized, high-resolution primate brain atlas may be feasible in the near future. TDat supports large data and thus may be highly useful in the reconstruction of primate neuronal circuits at single-neuron resolution. Its compatibility with various computing platforms renders TDat capable of handling even larger datasets in the future. We believe that TDat will be a powerful tool for neuroscience researchers.

## Author Contributions

QL and HG conceived the project. YL and AL designed the model and developed software. YL, HG, XY and QL wrote the article. JY, TJ and DZ produced the data sets. XL, QS prepared the specimens. YL and ZW tested software.

## Conflict of Interest Statement

The authors declare that the research was conducted in the absence of any commercial or financial relationships that could be construed as a potential conflict of interest.
