# Supplementary material for: TDat: An Efficient Platform for Processing Petabyte-Scale Whole-Brain Volumetric Images
Source: Front Neural Circuits. 2017 Jul 31;11:51. doi: 10.3389/fncir.2017.00051 (PMC5534480; doi:10.3389/fncir.2017.00051)
Supplement: Supplementary file 1 [file Data_Sheet_1.pdf]

Supplementary Material

TDat: An efficient platform for processing petabyte-scale whole-brain volumetric images

Yuxin Li[1,2], Hui Gong[1,2], Xiaoquan Yang[1,2], Jing Yuan[1,2], Tao Jiang[1,2], Xiangning Li[1,2], Qingtao Sun[1,2], Dan Zhu[1,2], Zhenyu Wang[1,2], Qingming Luo[1,2], Anan Li[1,2*]

[1]*Collaborative Innovation Center for Biomedical Engineering, Wuhan National Laboratory for Optoelectronics-Huazhong University of Science and Technology, Wuhan, Hubei 430074, China.*

[2]*Britton Chance Center and MOE Key Laboratory for Biomedical Photonics, School of Engineering Sciences, Huazhong University of Science and Technology, Wuhan, Hubei 430074, China.*

***Correspondence:** *Anan Li (aali@mail.hust.edu.cn).*

1 Supplementary Figures and Tables

1.1 Supplementary Figures

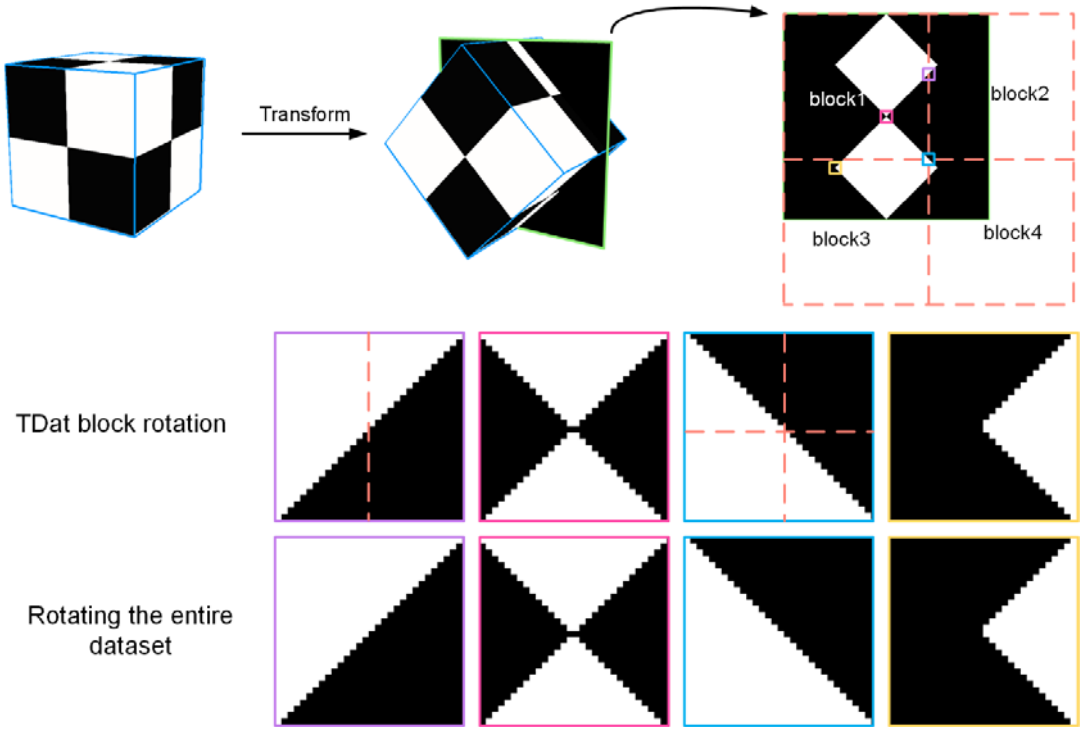

Supplementary Figure 1 | Comparison of the results between the TDat rotation and the rotation of the entire dataset.

An example of a 3DCM model data of size 512 × 512 × 512 px was rotated 45 degrees around the z-axis using two methods. Two slices with the same position in the transformed data were shown for comparison. Four regions were enlarged to show the detailed pixel value. The pink dotted line represents the borders of different 3D blocks in the TDat file.

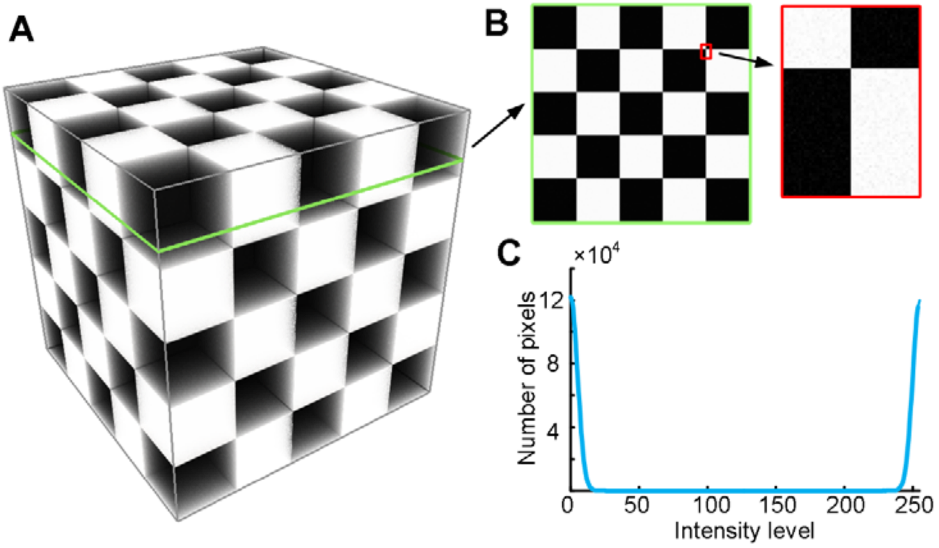

Supplementary Figure 2 | 3DCM model dataset.

(A) 3D visualization of a model dataset; the data size is 1,280 × 1,280 × 1,280 px; the size of each slice is 1,280 × 1,280 px with an 8-bit TIFF. The size of each black or white block is 256 × 256 × 256 px. (B) One slice in the 3DCM model dataset, and enlarged views of the red region. The Gaussian noise is shown. (C) The histogram of a slice in the model dataset, which reveals the 5 % Gaussian noise.

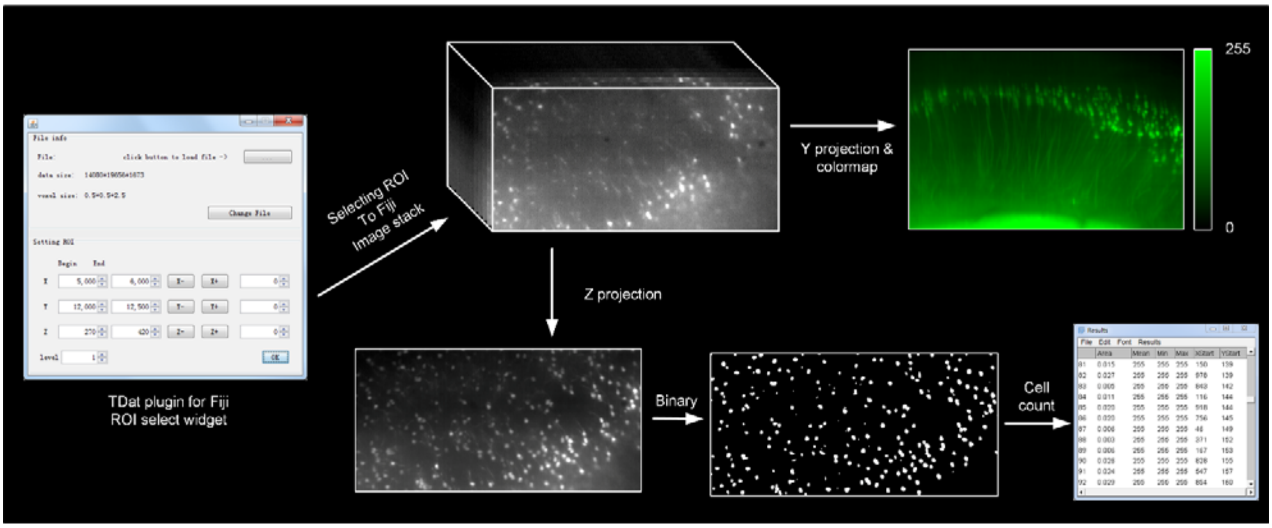

Supplementary Figure 3 | TDat-Fiji plugin for accessing TDat dataset in Fiji.

The TDat-Fiji plugin was used to read the ROI from the TDat dataset into the Fiji image stack, and Fiji was used to perform maximum intensity projections, image binary and cell counting. The ROI is a Thy1-EGFP F-line transgenic mouse hippocampus. The dataset was imaged using a light sheet microscope (UltraMicroscope II, Lavision Biotec GmbH) (see Dataset1 in **Table 2**).

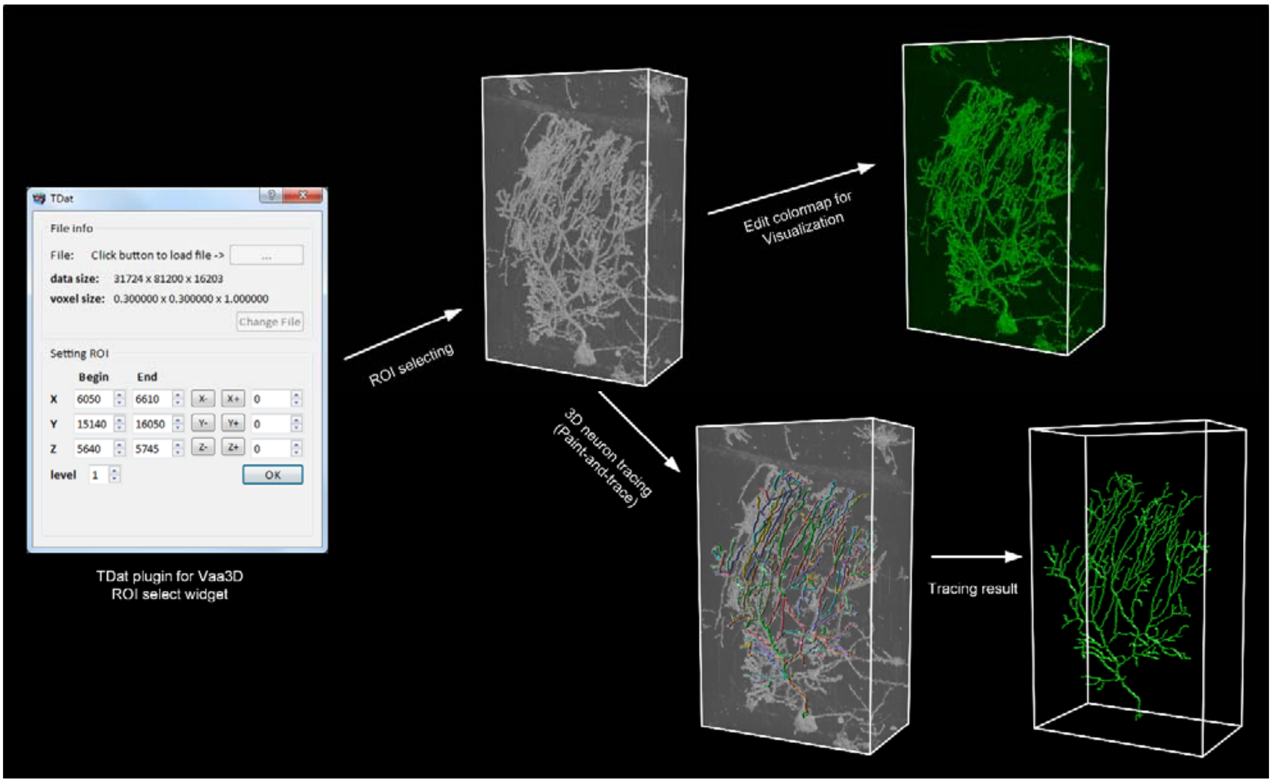

Supplementary Figure 4 | TDat-Vaa3D plugin for accessing a TDat dataset in Vaa3D.

The TDat-Vaa3D plugin was used to read the ROI from the TDat dataset into Vaa3D and edit the color map for visualization; paint-and-trace was used to trace neurons in 3D space. The ROI is an SD rat cerebellum. The dataset was imaged using a MOST (see Dataset4 in **Table 2**).

1.2 Supplementary Tables

Supplementary Table 1 Detail information of model dataset in this study reported here.

Image data	File size	Pixels	Bit depth	Noise	Reformatted size
**100 GB**	62.1 GB	4,753 × 4,753 × 4,753	8 bits	5 %	64.5 GB
**200 GB**	122 GB	5,989 × 5,989 × 5,989	8 bits	5 %	129 GB
**400 GB**	241 GB	7,545 × 7,545 × 7,545	8 bits	5 %	258 GB
**800 GB**	480 GB	9,506 × 9,506 × 9,506	8 bits	5 %	514 GB
**1,600 GB**	967 GB	11,977 × 11,977 × 11,977	8 bits	5 %	1 TB
**3,200 GB**	1.87 TB	15,090 × 15,090 × 15,090	8 bits	5 %	2.01 TB
**6,400 GB**	3.73 TB	19,012 × 19,012 × 19,012	8 bits	5 %	4.03 TB
**12.5 TB**	7.47 TB	23,954 × 23,954 × 23,954	8 bits	5 %	8.19 TB
**25 TB**	14.9 TB	30,180 × 30,180 × 30,180	8 bits	5 %	16.1 TB
**50 TB**	29.8 TB	38,024 × 38,024 × 38,024	8 bits	5 %	32.7 TB
**100 TB**	59.5 TB	47,908 × 47,908 × 47,908	8 bits	5 %	65.5 TB
**1 PB**	607 TB	104,032 ×104,032 ×104,032	8 bits	5 %	629 TB

Supplementary Table 2 Run time and memory usage for reformatting different size data between different methods.

**Image data**	**Amira-XLVolume**		**Fiji-bigdataviewer**		**TDat**		**Vaa3D-terafly**	
	Run time	RAM usage	Run time	RAM usage	Run time	RAM usage	Run time	RAM usage
**100 GB**	6.6 h	0.8 GB	5 h	16 GB [a]	0.36 h	1.4 GB	1.3 h	1.4 GB
**200 GB**	25 h	0.8 GB	10.2 h	16 GB [a]	0.91 h	1.6 GB	2.5 h	2.2 GB
**400 GB**	N/A	N/A	21.3 h	16 GB [a]	1.96 h	2 GB	5 h	3.6 GB
**800 GB**	N/A	N/A	N/A	N/A	4.06 h	2.5 GB	10.5 h	11.5 GB
**1,600 GB**	N/A	N/A	N/A	N/A	7.7 h	3.2 GB	21.1 h	18.3 GB
**3,200 GB**	N/A	N/A	N/A	N/A	12.7 h	4 GB	N/A	29.1 GB [b]
**6,400 GB**	N/A	N/A	N/A	N/A	24.8 h	5 GB	N/A	92 GB [b]

All benchmarks were repeated three times for each experiment, and they were run on a graphic workstation with 3.1GHz Intel E5-2687w×2 (16 cores), 192 GB of RAM, a disk array.

[a] The maximum heap size of Fiji was set to 16 GB.

[b] the value was given by Estimated RAM usage in Vaa3D-terafly.

N/A: We did not test because the computation time was excessive.

Supplementary Table 3 Run time for reformatting different size data on workstation and HPC.

	**Workstation**	**HPC**
**100 GB**	0.36 h	0.05 h
**200 GB**	0.91 h	0.07 h
**400 GB**	1.96 h	0.10 h
**800 GB**	4.06 h	0.17 h
**1,600 GB**	7.7 h	0.30 h
**3,200 GB**	12.7 h	0.55 h
**6,400 GB**	24.8 h	1.01 h
**12.5 TB**	N/A	1.97 h
**25 TB**	N/A	3.65 h
**50 TB**	N/A	7.15 h
**100 TB**	N/A	13.6 h
**1 PB**	N/A	144 h

All benchmarks were repeated three times for each experiment, the computers configuration are provided in **Table 3**.

N/A: We did not test because the computation time was excessive or the capacity of storage was limited.
